# Primary Pericardial Mesothelioma, Which Was Veiled by a Pleural Empyema: A Case Report and Review

**DOI:** 10.1155/2019/2896810

**Published:** 2019-09-11

**Authors:** Morad Tajjiou, Wolfgang Wild, Nasir Sayed, Alexander Flauaus, Markus Divo, Matthias Schwarzbach

**Affiliations:** ^1^Klinik für Allgemein-, Viszeral-, Thorax-und Gefäßchirurgie, Klinikum Frankfurt Höchst, Gotenstraße 6-8, 65929 Frankfurt am Main, Germany; ^2^Klinik für Radiologie, Neuroradiologie und Nuklearmedizin, Klinikum Frankfurt Höchst, Gotenstraße 6-8, 65929 Frankfurt am Main, Germany; ^3^Institut für Pathologie, Klinikum Frankfurt Höchst, Gotenstraße 6-8, 65929 Frankfurt am Main, Germany

## Abstract

This case report shows that pleural empyema limits the diagnostic significance of imaging techniques. Hereafter, we present the case of an 82-year-old patient with primary pericardial mesothelioma, which was veiled by a pleural empyema. The patient met the typical triad of signs of heart failure (dyspnea, lower leg oedema), pericardial effusion, and pericarditis. Echocardiography in the identification of pericardial mesotheliomas is low. In this case, the cardiac function could be imaged well, but the tumor could not be imaged. The CT showed a pericardial effusion and a pleural effusion. Here, the tumor could not be diagnosed either. Only the operation led to diagnosis.

## 1. Introduction

Primary pericardial mesothelioma is a highly malignant tumor and an oncologic rarity, with a prevalence of <0.002% [1]. When diagnosed, they are usually at an advanced stage. The average median survival time is extremely low, between 3 and 10 months after diagnosis. The most common causes of death are cardiac tamponade and heart failure [2]. Malignant pericardial mesotheliomas account for up to 50% of primary pericardial tumors. These patients often show nonspecific but typical symptoms like constrictive pericarditis, cardiac tamponade, and heart failure [2].

## 2. Case Report

We report about an 82-year-old patient who introduced himself to our emergency room. He complained of an increasing deterioration of his general condition, accompanied by the loss of appetite and a total weight loss of 8 kg in one month as well as an increase in stress dyspnea and increasing lower leg oedema on both sides for about a week. In addition, the patient reported about a stabbing thoracic pain occurring during inspiration. Previous diseases included arterial hypertension and hypothyroidism. The domestic medication consisted of L-thyroxine, candesartan, and hydrochlorothiazide (HCT).

Physical examination revealed a blood pressure of 118/82 mmHg, a heart rate of 82 beats/min, a respiratory rate of 18 breaths/min, a temperature of 37.1°C, and an oxygen saturation of 95% on 2 L of oxygen. Auscultation revealed a significantly reduced breathing sound on the left side and a vesicular breathing sound on the right side. The heart sounds were clear, rhythmic, and normofrequent; oedema of the lower leg appeared on both sides. An electrocardiogram showed SR, HF 95 bpm, bifascicular block, R/S envelope in V2, and T-negativation in V1. His blood chemistry revealed active inflammation (C-reactive protein 33.6 mg/dL, leukocyte 21.1 billion/L).

**Figure 1:**
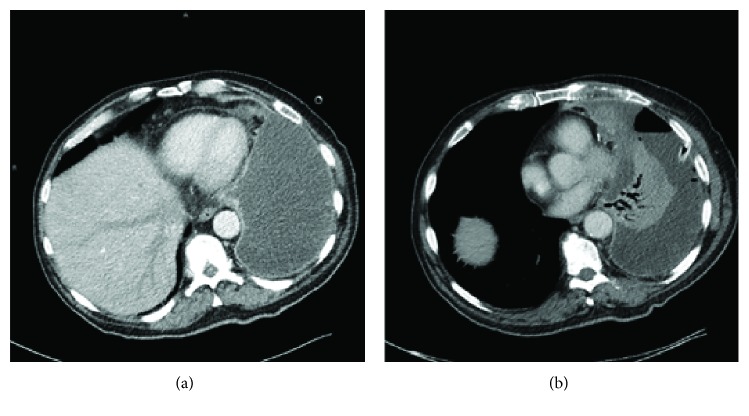
Computer tomography demonstrated (a) a pericardial effusion and (b) a pleural effusion.

A chest X-ray showed a complete shading of the left hemithorax with slight trachea deviation to the right. Transthoracic echocardiography (TTE) demonstrated a good systolic pumping function (LVEDD 38 mm, IVSD 14 mm) and circular pericardial effusion with maximum end-diastolic width of 0.8 cm. Computer tomography demonstrated an extensive pleural effusion on the left, filling in the entire left hemithorax and compressing the left lung tissue. A pericardial effusion with a maximum hem width at the tip of the heart of 1.9 cm was also observed (Figure 1).

Thoracentesis was carried out, and hemorrhagic pleural effusion was aspirated. The etiology of pleural effusion could not be identified because a cytological evaluation of the pleural fluid was negative for malignant cells. A bronchoscopy was performed with cryocanalization of some bronchial exits constricted by thickening of the mucous membranes. Biopsies of the left lung lower lobe were also obtained. Histologically, there was no evidence of malignancy. Clinically, there was a suspicion of a pleural empyema. Despite thoracic drainage and antibiotic therapy, high infection parameters in the laboratory and persistently high fever remained. The patient needed to be operated.

**Figure 2:**
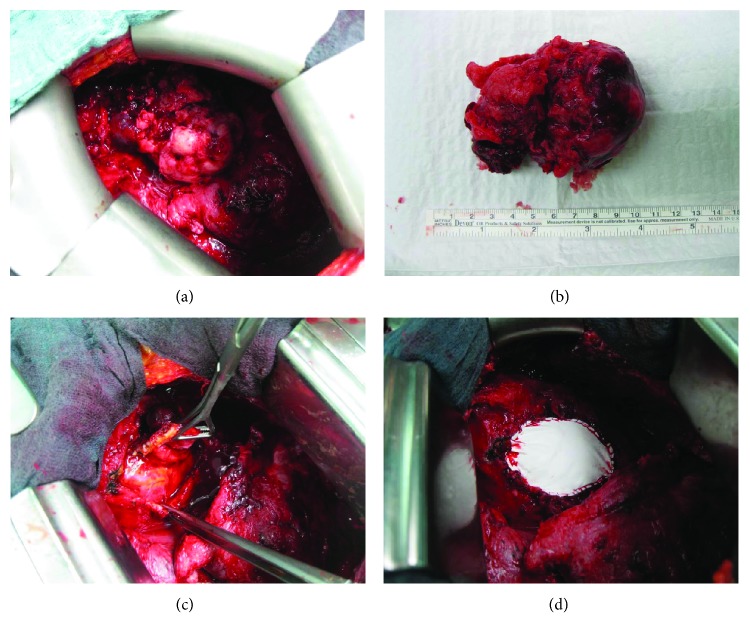
(a–c) The exploration revealed a 13 × 6 cm tumor originating from the pericardium and infiltrating the left ventricle wall. (d) The pericardial ventricle defect was sutured over; a pericardial patch (preclude pericardial membrane PCM Gore) was sutured into the pericardial defect continuously. In the distal part, a 15 × 15 mm gap was left as a pericardial window because of the preexisting pericardial effusion.

Thoracoscopy showed the typical picture of a grade III pleural empyema. During exploration, a 13 × 6 cm tumor was revealed, extending from the pericardium which infiltrated the left ventricle wall (Figures 2(a)–2(c)). An anterolateral thoracotomy was performed. The tumor was completely resected with the affected pericardial area (8 × 8 cm) and a small superficial area of the left ventricular wall (1 × 1 cm). A clear thickening of the pericardium to about 0.8 cm was observed. The ventricle defect was sutured over; a pericardial patch (preclude pericardial membrane PCM Gore) was sutured into the pericardial defect continuously. In the distal part, a 15 × 15 mm gap was left as a pericardial window because of the existing pericardial effusion (Figure 2(d)). This was followed by a further extensive decortication. The intraoperative instantaneous section provided a spindle cell tumor.

**Figure 3:**
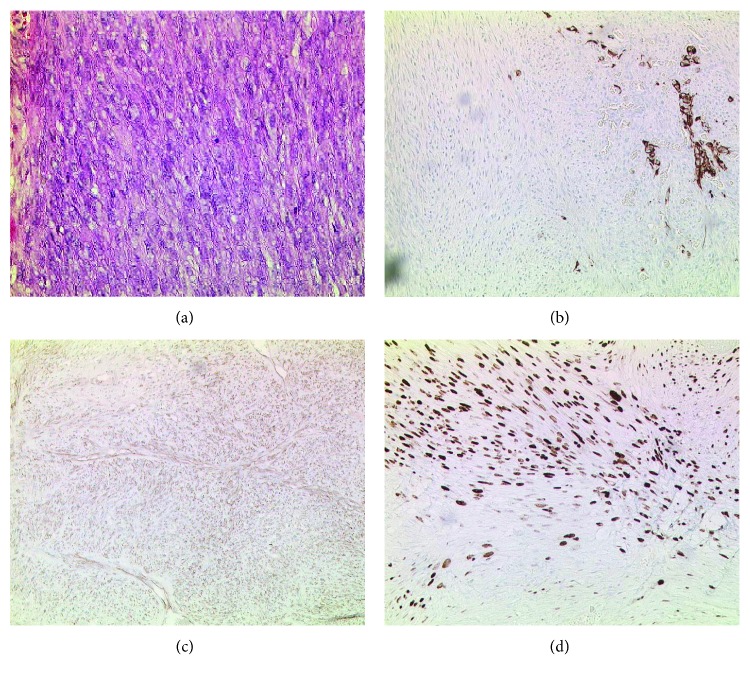
(a) Cell tight sections with core types and mitosis (HE color, 200-fold). (b) Immunohistochemistry with CAM 5.2 (cytokeratin 8/18), 100-fold, brown coloration = specific focal positivity. (c) Immunohistochemistry with calretinin, 100-fold, brown staining = diffuse positivity of tumor cells (typical marker for mesothelioma but may also be positive in other sarcomas and tumor entities). (d) Immunohistochemistry MIB-1, Ki67 equivalent, 100-fold, the brown cell nuclei mean positive reaction and indicate the proliferation index.

The patient recovered quickly and was transferred to the normal care unit after a short stay in our intensive care unit. The echocardiography controls showed a good pump function; the pericardial effusion was sufficiently drained through the pericardial window. According to an initial examination, the tumor was a highly malignant, predominantly spindle-shaped, microfocal epithelial cell tumor with perifocal metaplastic bone and cartilage formation, without evidence of a rearrangement of the SS18 gene in the complementary CISH. Based on the morphological image, we also considered the sarcoma, especially synovial sarcoma or a malignant peripheral nerve sheath tumor, for further subtyping of the malignancy. In synopsis, however, the morphological, immunophenotypic, and molecular-pathological findings, together with the clinical data, favor a predominantly sarcomoid, malignant mesothelioma, apparently starting from the pericardium (Figures 3(a)–3(d)).

“Next-generation sequencing” with the OCAv3 panel was used to detect mutations in tumor tissue in genes coding for the mTOR/p21 signaling pathway (TSC2 and PIK3R1), the NOTCH signaling pathway (NOTCH2), and the “G1/S DANN damage checkpoints” (CDKN2A and CDKN2B). Alterations in these signaling pathways are statistically significantly associated with pleural mesotheliomas [3]. The final examination showed a predominantly sarcomoid malignant mesothelioma originating from the pericardium. Chemotherapy with carboplatin and pemetrexed has begun. After appropriate supplementation with vitamin B12 and folic acid, the patient received the first dose six weeks after the surgical resection, which he tolerated well.

## 3. Discussion

Primary pericardial mesothelioma is a rare neoplasia with an incidence of <0.002% even among heart tumors and accounts for less than 5% of all mesotheliomas. Among primary, malignant pericardial tumors, pericardial mesothelioma is the most common primary pericardial tumor with a rate of 50%. However, metastases are much more frequent. The most common primary tumors for cardiac metastases are malignant melanoma, lymphoma, breast cancer, and lung cancer [4]. Primary pericardial mesothelioma is more common in men than in women (ratio 2 : 1 male to female). In contrast to pleural and peritoneal mesotheliomas, where there is an association with asbestos exposure, the cause of pericardial mesothelioma is not clear [5]. There was no asbestos exposure in our patient's prehistory. Other factors that may play a role are infection, radiation, nutritional factors, inflammation, genetic factors, or immunological impairment.

In several case reports, the most common form of clinical manifestation of pericardial mesothelioma was a triad of symptoms of cardiac insufficiency (due to cardiac tamponades, constrictive pericarditis, and in some cases myocardial infiltration), pericarditis that developed into constrictive pericarditis and pericardial effusion, for which diagnostic pericardial puncture was inconclusive. There are also case reports of primary pericardial mesotheliomas that manifested due to myocardial infiltration or of brain embolisms (cerebral embolisms) that caused neurological deficits [2, 6]. Primary pericardial mesotheliomas are highly malignant tumors, and when diagnosed, they are usually at an advanced stage. The average median survival time is extremely low, between 3 and 10 months after diagnosis. The most common causes of death are cardiac tamponade and heart failure [2]. In this case, the patient met the typical triad of signs of heart failure (dyspnea, lower leg oedema), pericardial effusion, and pericarditis, which was only seen intraoperatively. In addition, the patient presented a septic constellation with fever, high infection parameters in the laboratory, and a pleural empyema. The diagnostic pleural puncture was also not significant in our case. Besides echocardiography, imaging methods (CT, MRI) are important diagnostic tools. However, the sensitivity of echocardiography in the identification of pericardial mesotheliomas is low [7]. In this case, the cardiac function could be imaged well, but the tumor could not be imaged. The CT showed a pericardial effusion and a chambered pleural effusion (Figure 1). Here, the tumor could not be diagnosed either. In this case, the operation led to the diagnosis. A curative resection is possible with small tumors [8]. But in most cases, only a palliative therapy is possible at the time of diagnosis. Surgical intervention in pericardial mesothelioma is primarily for cytoreduction before multimodal therapy or to deliver and monitor innovative intrapericardial therapies. In our case, we found a 13 × 6 cm pericardial tumor infiltrating the left ventricle wall (Figure 2(c)). In addition, tumor cells were found in the pleura as a sign of pleural metastasis. Whether the tumor promoted pleural empyema or whether it can be regarded as a separate symptom cannot be clarified conclusively. However, it shows that a pleural empyema significantly limits the diagnostic significance of imaging techniques.
